# CuO/rGO doped with silver nanoparticles for humidity sensing applications

**DOI:** 10.1039/d5ra01883h

**Published:** 2025-05-07

**Authors:** Amir Elzwawy, Mohamed Morsy, Sara Zain, Ahmed I. Abdel-Salam

**Affiliations:** a Ceramics Department, Advanced Materials Technology and Mineral Resources Research Institute, National Research Centre (NRC) 33 El Bohouth St., Dokki Giza 12622 Egypt elzwawy1@gmail.com aa.elzwawy@nrc.sci.eg; b Faculty of Engineering and Environment, Northumbria University Newcastle Upon Tyne NE1 8ST UK; c Nanotechnology Research Centre (NTRC), The British University in Egypt (BUE) Suez Desert Road, El-Sherouk City Cairo 11837 Egypt; d Building Physics and Environment Institute, Housing & Building National Research Center (HBRC) 12311 Dokki Giza Egypt

## Abstract

Determination of the relative humidity in the surrounding environment is essential for numerous industrial and technological applications. In this work, we successfully prepared CuO–rGO doped with varied Ag concentrations (0–1.5 wt%). The XRD measurements demonstrated that the structures were successfully developed with an average crystallite size of 30–40 nm as reflected from the (111) plane, with a dominating cubic phase. The SEM morphological characteristics demonstrated that the cubic structure was Cu-based, whilst the sheet-like structure was attributable to 2D rGO. The cubic structure tended to lose its regular shape, while the size tended to be reduced as the Ag doping ratio increased. Elemental analysis was confirmed through EDX for CuO–rGO doped with 1.5 wt% Ag, reflecting 35, 13, 1.4, and 50.6 wt% of C, O, Ag, and Cu, respectively. Assessment of the antimicrobial assets of the nanostructures *versus* G^+ve^ (*Staphylococcus*), and G^−ve^ bacteria (*Escherichia coli*) presented the highest activity for CuO–rGO doped with 1 wt% Ag. The humidity sensing evaluations were revealed throughout a wide set of frequencies (50–10 kHz) and humidity levels (11–97% RH). The optimum frequency was optimized as 50 Hz. The acquired response and recovery times were 154, and 172 s, respectively, while the sensitivity was 2 × 10^6^ Ω per RH for CuO–rGO doped with 1.0 wt% Ag. Remarkably, the recovery time for CuO–rGO doped with 1.5 wt% Ag was 17 s. The sensor demonstrate decent repeatability for four cycles between 11% and 75% RH at a testing frequency of 50 Hz. The results nominate this structure as an affordable, low-cost, and applicable humidity sensor valid for nanotechnological and materials science routes.

## Abstract 2

Determination of the relative humidity in the surrounding environment is essential for numerous industrial and technological applications.

## Graphical abstract

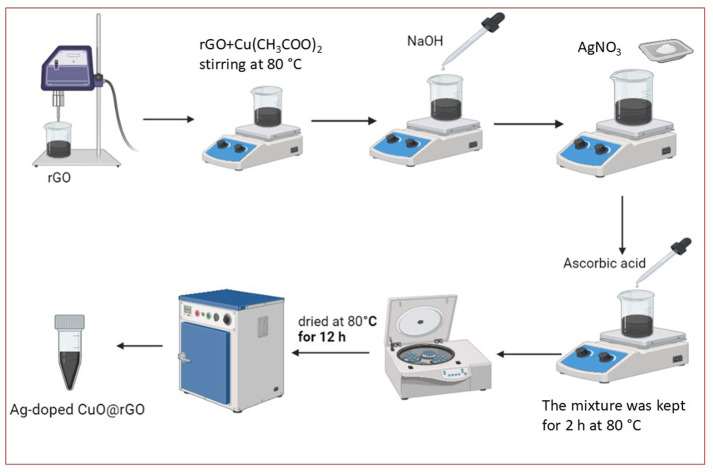

## Introduction

Industrialization and modern life requirements have increased the necessity for feasible approaches to produce industrial applications demanded by mankind's daily activities.^1–4^ The tracking and adjustment of humidity are imperative in agriculture, environmental conservation, industrial and medical sectors.^5–7^ Innovative humidity sensors constructed on semiconducting metal oxides with decent humidity-sensing features have invoked widespread consideration, owing to their elevated room temperature sensitivity, raised safety, reduced hysteresis magnitude, and long-term stability.^1,8^ These sensors deliver significant information by assessing the moisture amount in the surrounding air humidity, which is critical for sustaining optimum situations in several settings.^9^ Researchers and engineers are actively involved in developing desirable sensing devices with tailored features.^10–12^ These features should comprise a cost-effective approach, effective response and recovery duration, compatibility, durability and operating ranges, and the feasibility of functionalization.^6,13^ Graphene oxide (GO) is denoted as a 2D material attained from the graphitic structures' oxidation, and it holds a blend of diverse functional oxygen groups.^14,15^

Recently, graphene oxide and its derivatives have attracted considerable attention in numerous fields owing to the simplicity of fabrication and potential for tailored specifications.^14,16–18^ Inherently, the decoration and conjugation between graphene oxide and other metal oxides are a valid approach for constructing improved performance sensors.^19–21^ Thereafter, the employment of rGO is favored due to the enriched conductive sp^2^ carbon atoms, which behave as anchors to initiate the electron transfer in proximal metal oxides.^22^

The hybridization between the rGO and CuO is reported for different applications.^23^ For instance, supercapacitors and energy storage,^24,25^ sensing,^26–28^ optoelectronics,^20,29^ and catalysis.^30^

Sreejivungsa *et al.*^31^ explored the capacitive humidity sensing assets of CuO ceramic. CuO capacitance continuously increases with rising RH levels, from 30% to 95%. The CuO ceramic verified a fast response and recovery times of around 2.8 and 0.95 min, respectively. Zhenyu Wang *et al.*^28^ fabricated a humidity sensor based on the CuO/rGO composites and showed that it delivered increased impedance compared with sole CuO or rGO. This elevation in the impedance arises from the contribution of the impedance by Schottky junctions when CuO is well coupled with rGO.^28^ Yu *et al.*^21^ provided triboelectric nanogenerators driven by a self-powered rGO–TiO_2_ humidity sensor which shows elevated response/recovery times (1/5.2 s) and limited humidity hysteresis (0.54%) with a stable repeatability. Li *et al.*^32^ developed a humidity sensor that relied on GO–Ag nanoparticles, with the best performance obtained with the GO/Ag (2 wt%) composite-based sensor throughout 11–97% RH with a 8/12 response and recovery durations.

The ternary nanocomposite of rGO–CuO-Ag is less reported in the literature. Akhalakur Rahman Ansari *et al.* synthesized this structure and applied it in as a supercapacitor.^33,34^ Besides, this structure is also introduced for the catalytic activity and antimicrobial properties.^35,36^ Sridevi *et al.*^37^ explored optoelectronics based on the ternary rGO–CuO doped Ag.

As far as the authors know, this ternary nanocomposite of rGO–CuO doped Ag nanoparticles is not reported in the humidity sensing application. In this research, the authors have synthesized the ternary nanostructures of rGO–CuO doped with Ag nanoparticles (0, 0.5, 1.0, and 1.5 wt%). The structural, morphological, and physical nature of the structures were inspected through XRD, FTIR, SEM, and EDX. The antimicrobial properties of the structures were also explored. Finally, the proposed structure is applied in humidity sensing through a wide scale of environmental humidity levels. The introduced structure provides helpful insights for humidity sensing which is beneficial for a wide set of industrial and nanotechnological applications.

## Experimental

### Materials

The starting materials and precursors were used as it is, without any treatment or purifications. Copper acetate Cu(CH_3_COO)_2_ of 98% purity and sodium hydroxide of 97% purity were purchased from Sigma-Aldrich. Silver nitrate (AgNO_3_) of 99.9% purity was supplied by Sigma-Aldrich. The GO was synthesized using the modified hummer method. Milli Q deionized water was used as a solvent in all experiments. The details regarding the synthesis process can be found in our previously published articles.^38–40^ All chemicals have been used as is without any further treatment or purification.

### Synthesis of CuO@rGO doped with Ag nanoparticles

**Fig. 1:**
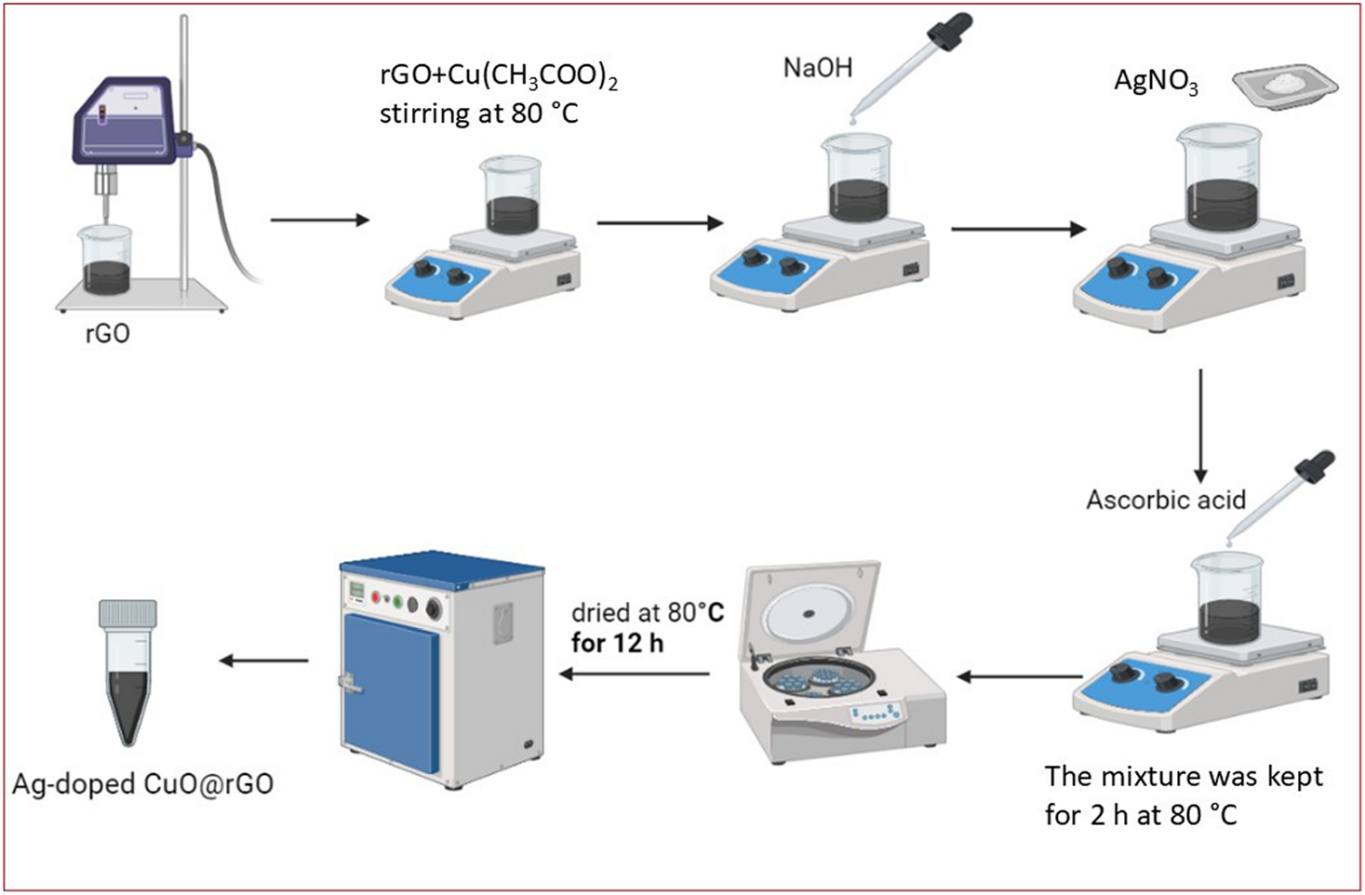
Schematic of the synthesis procedure of CuO@rGO doped with Ag nanoparticles.

CuO@rGO doped Ag nanoparticles was prepared using the co-precipitation method. First, the GO was dispersed in 50 mL of DI water using the probe sonicator for 10 min. After that, 1 g of Cu (CH_3_COO)_2_ was added to the GO dispersion with continuous stirring at 80 °C, followed by dropwise addition of 1 M NaOH. After complete addition of NaOH, diverse AgNO_3_ concentrations were added to the mixture and delivered as (0, 0.5, 1, and 1.5 M) to study the composite's structure, morphological properties, and sensing performance at different levels of Ag doping, followed by dropwise addition of 0.1 M ascorbic acid. The reaction mixture was kept for 2 h at 80 °C. Finally, the produced precipitate was separated and washed several times with DI water using centrifugation at 8000 rpm, followed by drying the precipitate at 80 °C for 12 h. The schematic diagram of the synthesis process is illustrated in Fig. 1.

### Characterization techniques

The synthesized materials were investigated using different techniques to explore their properties and confirm their physical and chemical properties. The crystal structure was investigated using XRD (Malvern Panalytical Empyrean 3 diffractometer) with a Cu Kα radiation source (*λ* = 1.5406 Å). Morphological features were revealed by the SEM/EDX instrument (Thermo Fisher Scientific), operated at 20 kV. The interactions of the functional groups and bands were explored through FTIR (Vertex 70, Bruker) in a spectral range of 4000–400 cm^−1^, maintaining a 4 cm^−1^ spectral resolution.

### Humidity sensing set-up and testing

The humidity sensor was fabricated using a simple cast method technique. A suitable amount of sensing material was ground in an agate mortar for 5 min; then, a minimal amount of de-ionized water was added to the sensing materials and grounded again to form a slurry. The formed slurry was deposited over a pre-cleaned FTO substrate and allowed to dry at 60 °C for 24 h. The humidity sensing performance of the prepared samples was investigated between 11% up to 97% humidity at room temperature. A fixed level of humidity was attained using the saturated salt solution in a closed conical. The saturated salt solution generates a specific level of humidity based on the type of salt. The desired level of relative humidity was generated using the guidelines of ASTM E104 “Standard Practice for Maintaining Constant Relative Humidity by Means of Aqueous Solutions”. The investigated sensor was aged for 24 h before any evaluation at low and high humidity to stabilize the output signal and reduce the signal-to-noise ratio. The sensor was clipped with two alligators and connected to LCR (HIOKi 3532-50) bridge to measure the impedance variation as a function of humidity level. The sensor was stabilized for 15 min at each humidity level, and then the corresponding impedance value was recorded.

### Antibacterial assays

The antibacterial assays were established using two dissimilar methods according to CLSI.^41^ These methods are disk diffusion (Kirby–Bauer)^42^ and absorbance measurements at OD 600 nm. For the disk diffusion method, *Staphylococcus aureus* (ATCC 6538) and *Escherichia coli* (ATCC 8739) were employed to represent G^+ve^ and G^−ve^ bacteria, respectively.

## Results and discussion

### XRD

**Fig. 2:**
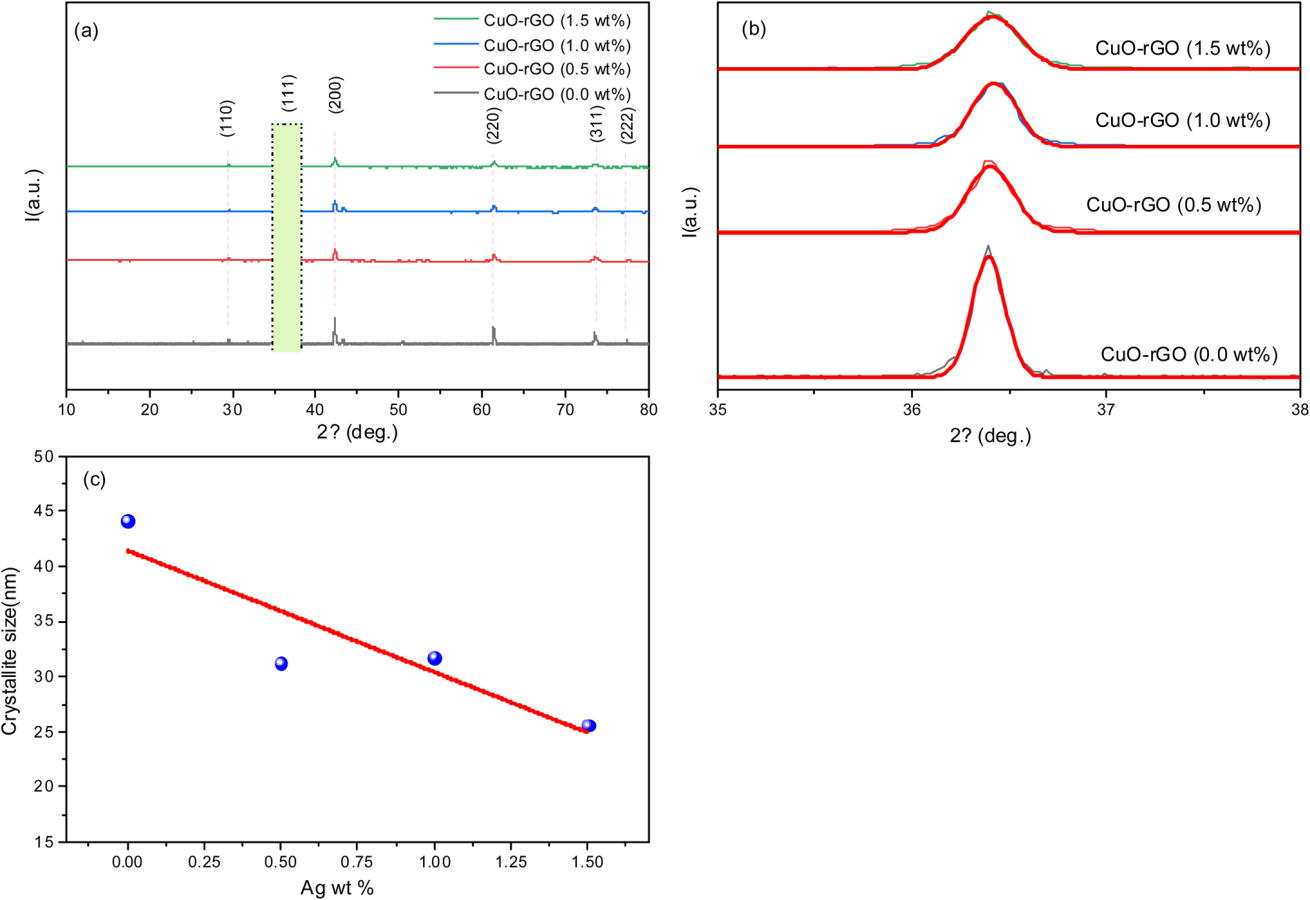
(a) X-ray diffraction pattern of the synthesized nanocomposite of CuO–rGO (doped with 0–1.5 wt% Ag). (b) Gaussian fitting of the highest intense peak resident around 36°, highlighted in (a). (c) Representation of the crystallite size with Ag wt% accumulation.

X-ray diffraction is an essential tool for reporting the key crystallographic features of the prepared nanocomposites. In this work, we have synthesized CuO–rGO doped with minor traces of Ag (0–1.5 wt%). At first glance, the diffraction pattern reveals a crystalline structure reinforced by the emergence of intense peaks with a narrowed full width at half maximum (Fig. 2). Cuprous oxide (Cu_2_O) dominates the structure and has diffraction peaks around 29.5°, 36.4°, 42.3°, 61.3°, 73.5°, and 77.3°, which were clearly observed throughout the pattern (Fig. 2a); these peaks are indexed to (110), (111), (200), (220), (311), and (222), respectively. These results coincide with ICSD card no. 01-080-7711 with a negligible shift (<1°) and former reports.^43,44^ Cu_2_O has a cubic structure (*a* = *b* = *c* = 4.27 Å, *α* = *β* = *γ* = 90°). Characteristically, graphene oxide has a dominant peak around 11° allocated to (001) plane,^45^ while the absence of this peak clarifies the successful reduction of the graphene oxide as elucidated in the presented structures. Further, the disappearance of the rGO peaks might be attributed to the anchoring of CuO nanoparticles on the rGO surface hindering rGO stacking.^46^ The silver nanoparticles reduced wt% in the network does not reflect any high intense peaks as expected; however, silver has the ICSD card no. 01-087-0597, with peaks around 38.1° and 44.2° for the (111) and (200) planes, accordingly. The disappearance of these sharp peaks might arise from the tiny amount compared to the major amount of cuprous oxide. The inclusion of silver nanoparticles has an impact on the crystallinity degree due to the replacement of Cu with Ag. They have slightly differed atomic radii; thus, there is an expected dislocation density, microstrain, and interruption in the periodic nature of the structure. The highest peak intensity (∼36°) tends to decrease intensity upon Ag doping elevation (Fig. 2b).

The crystallite sizes of the structures are determined using the Scherrer equation, while the most intense peak located at ∼36° is assigned for the calculation. The Scherrer equation is introduced mathematically^47^ according to eqn (1):1
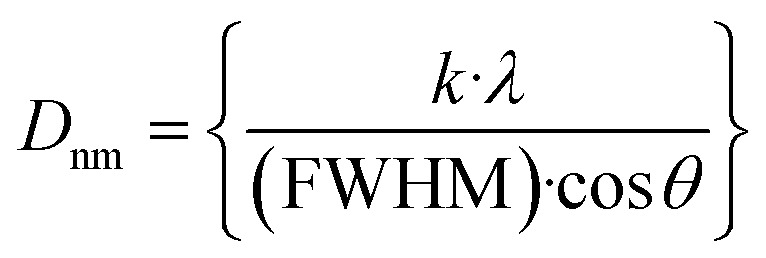


The parameters in the preceding equation are the shape factor (*k*), having a nominal value around 0.9, the X-ray incident wavelength for the Cu Kα source (*λ*), and Bragg's angle for the diffraction. Key parameters of the structures were calculated, including dislocation density, microstrain, and theoretical density.^6–8,48–50^2
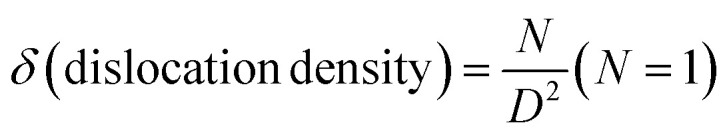

3 *ε*(microstrain) = (FWHM/4 tan *θ*)

The theoretical density (*D*_theo._) of the synthesized nanostructures is further considered by employing the molecular weight (*M*_W_), Avogadro's no. (*N*_A_), and unit cell volume (*V*) as follows:^51^4
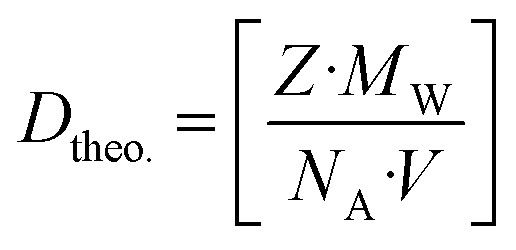


*Z* represents the parameters formula with a magnitude of 8. Besides, the specific surface area (S. A.) is associated with the theoretical density as follows:^51^5
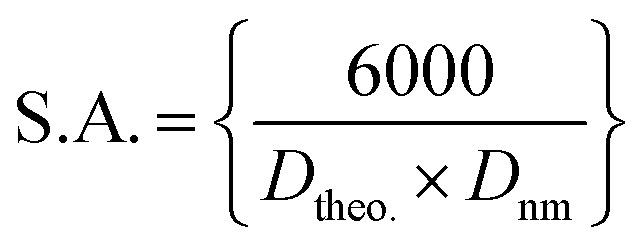


The outcomes of the XRD parameters are tabulated in Table 1. The incorporation of silver nanoparticles into the network of the structure yields an elevated particle size and specific surface area for the lowest concentration along with the lowest theoretical density. Apparently, the cubic structure tends to misplace its regular shape, while the size tends to be reduced as the Ag doping ratio increases.

**Table 1:** XRD parameters of CuO–rGO (doped with Ag 0–1.5 wt%)

Composition	*D* (nm) Scherrer equation	FWHM (deg)	*δ* (nm^−2^)	*ε*	*D*_theo._ (g cm^−3^)	SA (m^2^ g^−1^)
CuO–rGO-Ag 0.0	44.13	0.198	5.13 × 10^−4^	26.274 × 10^−4^	3.98	20.4
CuO–rGO-Ag 0.5	31.23	0.2798	10.25 × 10^−4^	31.569 × 10^−4^	4.13	22.3
CuO–rGO-Ag 1.0	31.77	0.2750	9.91 × 10^−4^	20.233 × 10^−4^	4.2	23.1
CuO–rGO-Ag 1.5	25.59	0.3414	15.27 × 10^−4^	19.958 × 10^−4^	4.26	24.2

### SEM observations

**Fig. 3:**
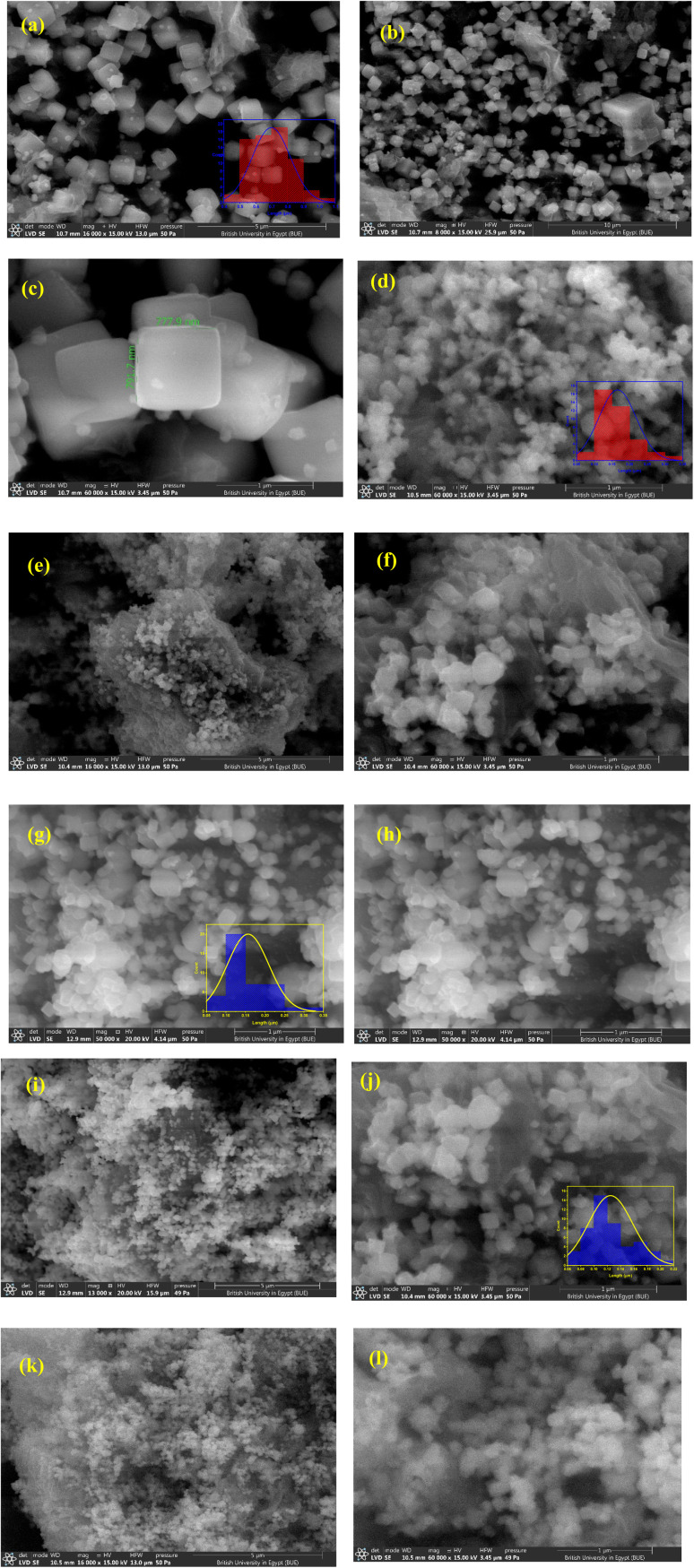
SEM images of the synthesized materials [(a)–(c)] for CuO/rGO, [(d)–(f)] for CuO/rGO-0.5 M Ag, [(g)–(i)] for CuO/rGO-1 M Ag, and [(j)–(l)] for CuO/rGO-1.5 M Ag. The histograms are displayed as insets in (a), (d), (g) and (j) for CuO/rGO, CuO/rGO-0.5 M Ag, CuO/rGO-1.0 M Ag, and CuO/rGO-1.5 M Ag, respectively.

SEM was used to observe the morphological features of the prepared materials while the elemental analysis was confirmed by EDX analysis. The SEM images of the Cu/rGO sample (Fig. 3a) revealed two different constituents in the structures. The cubic structure was CuO, while the sheet-like structure was a 2D rGO.

The synthesized materials were examined using SEM, while the elemental analysis was confirmed by EDX analysis. The SEM images of Cu/rGO sample (Fig. 3a) reveal two different constituents in the structures. The cubic structure is CuO, while the sheet-like structure is a 2D rGO. The CuO cubes have sharp edges with approximately the same size. The effect of Ag addition at 0.5 M, 1 M, and 1.5 M was explored in Fig. 3b–d, respectively. The SEM images demonstrate that Ag has a direct effect on the cubic structure of CuO. The cubic structure tends to lose its irregular shape while the size tends to be reduced. The average estimated lengths of the CuO cubic structure were 0.7 μm, 0.16 μm, 0.16 μm, and 0.12 μm for 0 M, 0.5 M, 1 M, and 1.5 M Ag addition, respectively using Image J software.

**Fig. 4:**
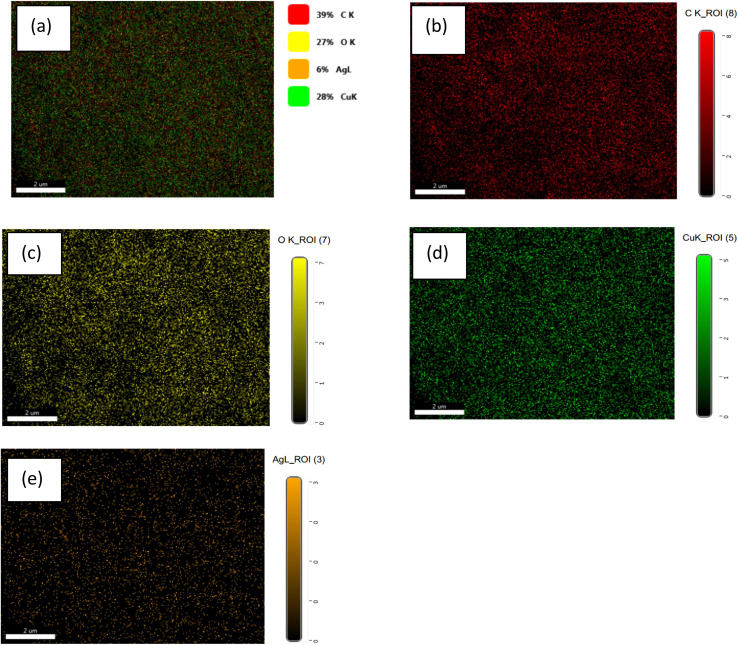
Mapping elemental analysis distribution of CuO/rGO-1.5 M Ag (a), showing the elements C (b), O (c), Cu (d), and Ag (e).

The presence and distribution of different elements in the synthesized materials were confirmed through EDX and elemental mapping analysis. EDX mapping analysis conducted for CuO/rGO-1.5 M Ag is shown in Fig. 4. The EDX analysis (Fig. 4 and Table 2) confirmed the presence of copper, oxygen, carbon, and silver as main components of the investigated sample (Table 2). Mapping analysis revealed the presence and distribution of all elements in the synthesized material (Fig. 4a). The individual distributions of carbon, oxygen, copper, and silver are indicated in Fig. 4b–e, respectively. The mapping analysis confirmed the homogeneous distribution of Ag inside the main matrix, as indicated in Fig. 4e.

**Table 2:** EDX elemental analysis of CuO/rGO-1.5 M Ag

Element	Weight %	Atomic %	Net Int.	Error %
C K	34.96	64.14	252.74	8.80
O K	13.09	18.03	129.36	8.97
Ag L	1.34	0.27	10.00	17.67
Cu K	50.62	17.55	116.78	3.89

### FTIR

**Fig. 5:**
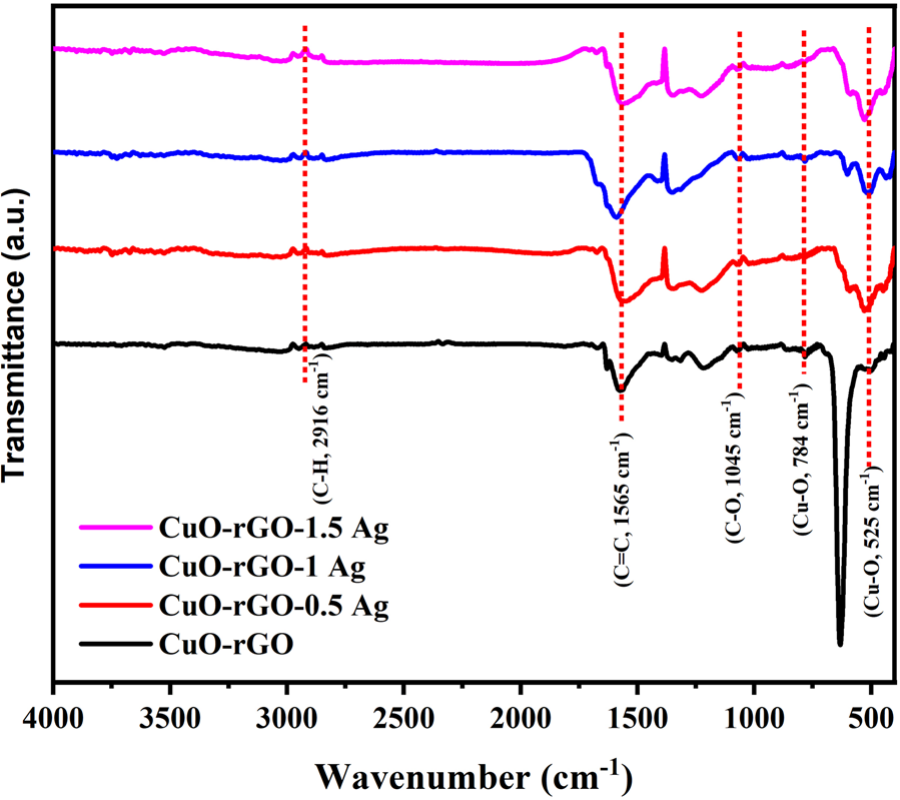
FTIR spectra of the acquired nanostructures.

The FTIR spectra of the synthesized structure are shown in Fig. 5. The FTIR spectra were recorded using KBr disk method from 400 cm^−1^ up to 4500 cm^−1^. The CuO–rGO sample exhibited a very weak transmittance peak at 3452 cm^−1^ that was attributed to O–H stretching. Usually, the intensity of O–H stretching peak is associated with the reduction degree, the less intense the peak, the more reduced graphene oxide. The very weak peak is a good indicator for successful reduction of GO. The CuO–rGO sample exhibited characteristic peaks at 1565 cm^−1^ and 1045 cm^−1^ which are corresponding to the C=C and C–O vibration modes of the rGO, respectively. In addition, the vibrational modes that appear at 784 cm^−1^ and 525 cm^−1^ are characteristic for the Cu–O this confirm that the CuO–rGO has been successfully prepared. The peak at 625 cm^−1^ belongs to the vibration of Cu–O.^52,53^ A broad peak was recognized for CuO–rGO-1.0 Ag. The OH groups can serve as adsorption sites for polar gases (*e.g.*, alcohols, NH_3_), enhancing the sensitivity of the sensor.^54^ The surface hydroxylation can modulate the electronic properties of the composite, influencing charge transfer mechanisms and impacting the sensor's electrical response.^55^ Thus, Ag nanoparticles further facilitate rapid electron transfer due to their high conductivity, amplifying the signal generated upon gas interaction.^56^ The pronounced OH signature observed in the Ag-doped CuO/rGO composite likely arises from increased surface hydroxylation facilitated by the presence of Ag nanoparticles. Ag presents additional active sites and alters the surface polarity and defect structure, making the surface more favorable for OH group adsorption. This enhanced hydroxylation is beneficial for sensing applications as it improves surface reactivity, electron transfer, and ultimately enhances the sensitivity and responsiveness of the sensor.

### Humidity sensor evaluation

**Fig. 6:**
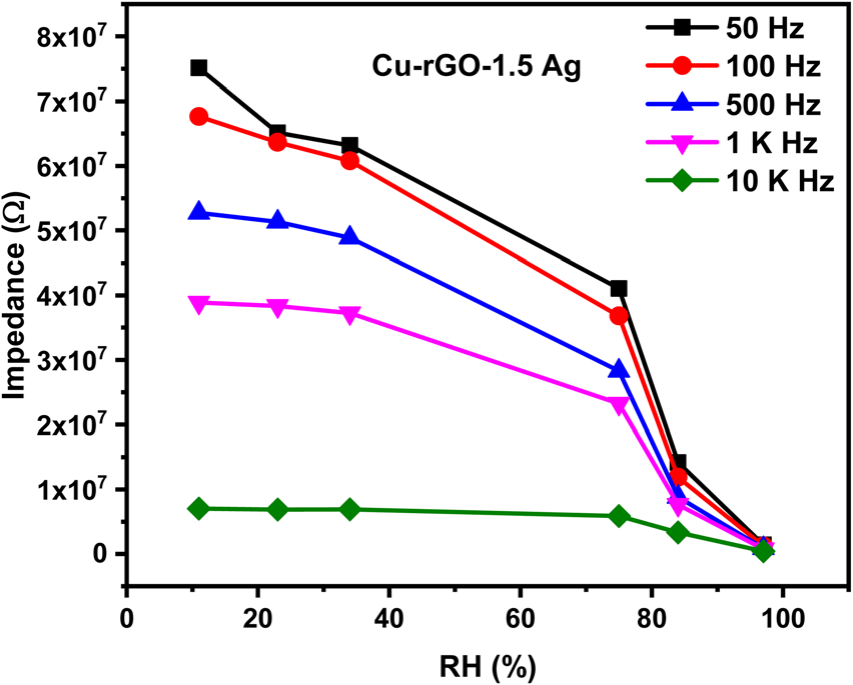
Variation in impedance magnitudes of CuO–rGO-Ag 1.5 wt% with humidity level propagation at different frequencies.

The fabricated sensors were evaluated at different humidity levels, where the impedance variation as a function of relative humidity levels at different testing frequencies is shown in Fig. 6. The main target of this test is to determine the optimum testing frequency. The polarizability of adsorbed water molecules is affected by the applied frequency; as the frequency increases, the adsorbed water molecules were unable to follow the alternation in the applied frequency. This explains why the impedance variation is insignificant at higher testing frequencies. The results proved that 50 Hz is the optimum testing frequency for the sensor, which was used in further experiments.

**Fig. 7:**
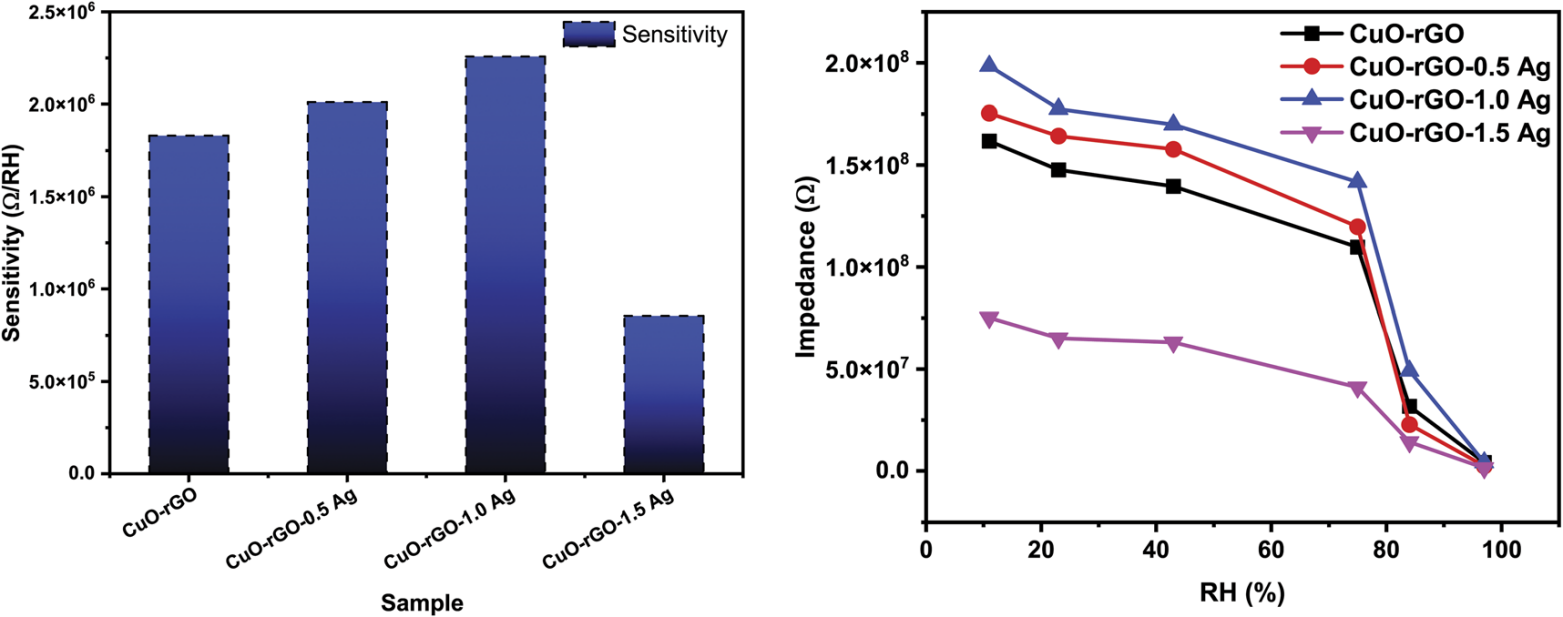
(Left) Sensitivity values of the prepared structure represented in columns for easier estimation. (Right) Propagation of the impedance with various humidity levels at the optimized frequency of 50 Hz.

The impedance variation is shown as a function of relative humidity level curves for all examined sensors in Fig. 7. All investigated sensors revealed a decrease in impedance as the relative humidity level increased from 11% up to 97%. The sensitivity of the humidity sensor is defined as the change of impedance per humidity level change in the following equation:^57^6
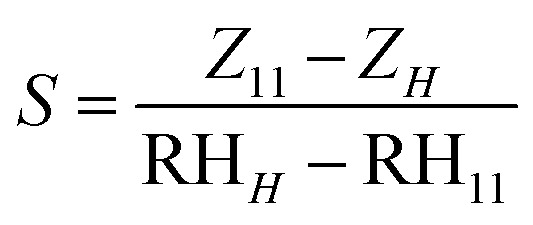
where *Z*_11_ and *Z*_*H*_ are impedance values at 11% RH and 97% RH, respectively. The sensitivity of all measured sensors is shown in Fig. 7. The impedance increases as the amount of Ag increases up to 1 M, then decreases. Therefore, CuO–rGO-1.0 Ag demonstrated the highest humidity sensing sensitivity among all the tested sensors.

**Fig. 8:**
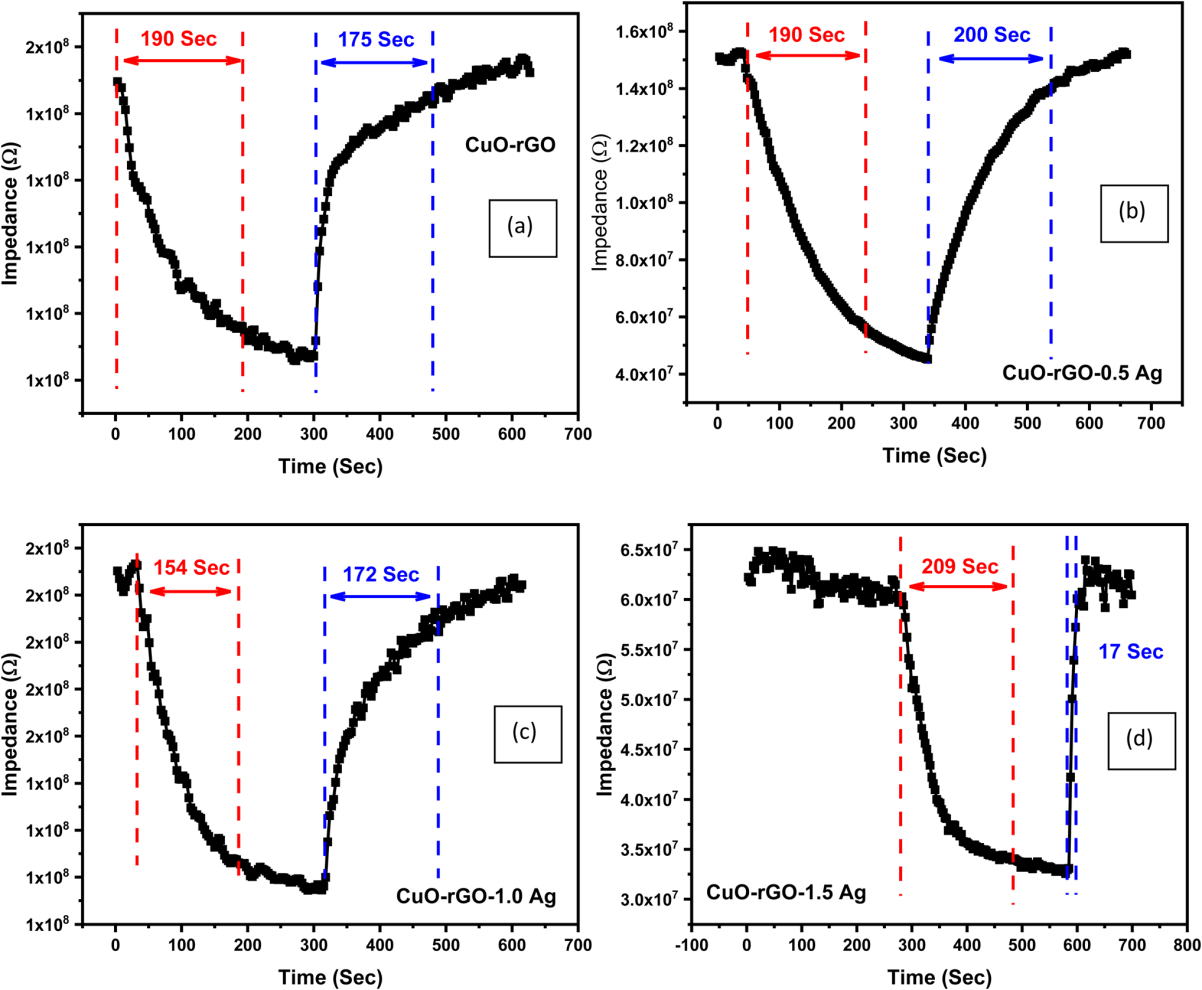
The response and recovery times of the synthesized nanostructures. (a) CuO–rGO, (b) CuO–rGO 0.5 Ag, (c) CuO–rGO 1.0 Ag, and (d) CuO–rGO 1.5 Ag.

The response and recovery times are one of the most important factors that deserve more attention when focusing on humidity sensing responses of a given material. The response time is defined as the time required by the sensor to reach 90% of its minimum impedance value, while the recovery time refers to the time taken by the sensor to reach 90% of its baseline value. The response and recovery times of CuO–rGO, CuO–rGO-0.5 Ag, CuO–rGO-1.0 Ag, and CuO–rGO-1.5 Ag sensors is demonstrated in Fig. 8a–d. The response and recovery time improved due to the addition of Ag, while the best performance was attained for the CuO–rGO-1.0 Ag sensor. Although the CuO–rGO-1.5 Ag sensor demonstrated the fastest recovery than the other sensors, the response time was higher. It was concluded that the CuO–rGO-1.0 Ag sensor exhibited exceptional performance compared to the studied sensors based on the measurements of the performance evaluating parameters.

**Fig. 9:**
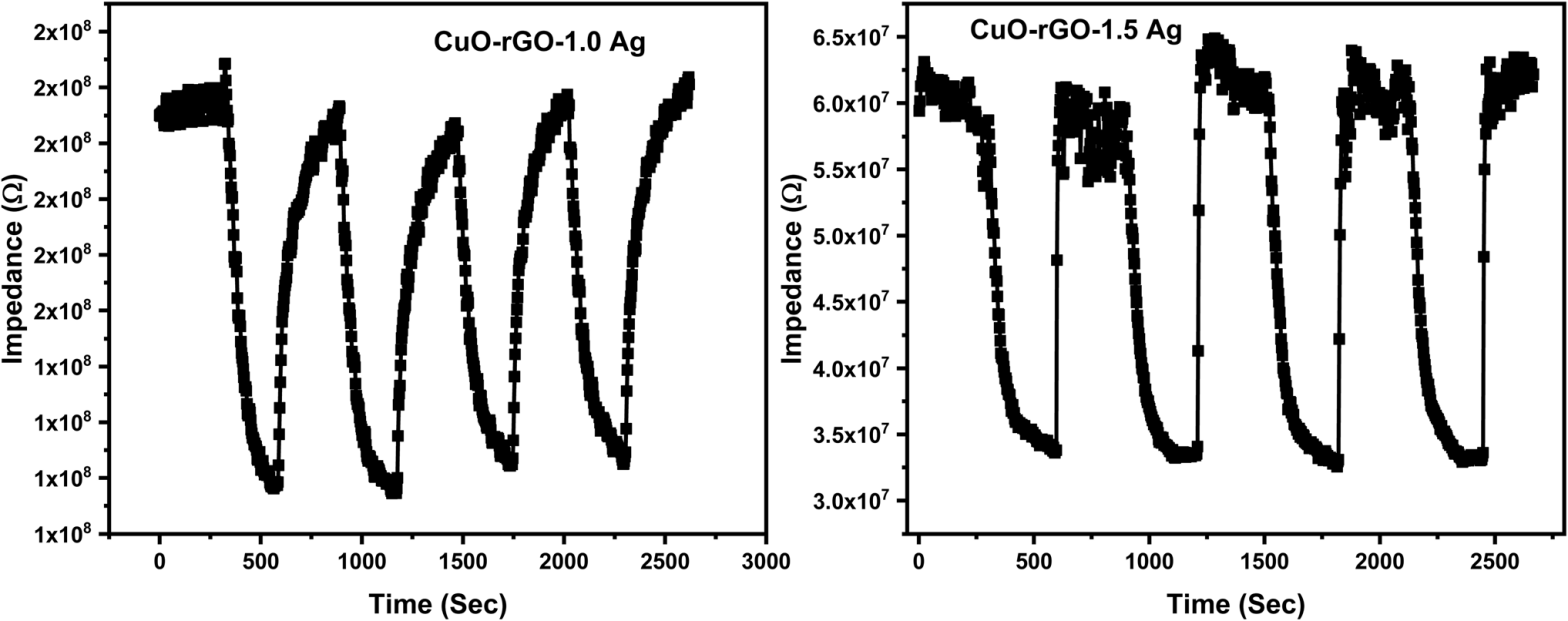
Repeatability of the prepared structures of CuO–rGO doped with 1.0 Ag and 1.5 Ag nanoparticles showing the stability of the performance.

The repeatability of CuO–rGO-1.0 Ag and CuO–rGO-1.0 Ag are revealed in Fig. 9. The sensors were investigated for four cycles between 11% and 75% at testing frequency of 50 Hz. The investigated sensors revealed a good repeatability over the measured relative humidity levels.

**Fig. 10:**
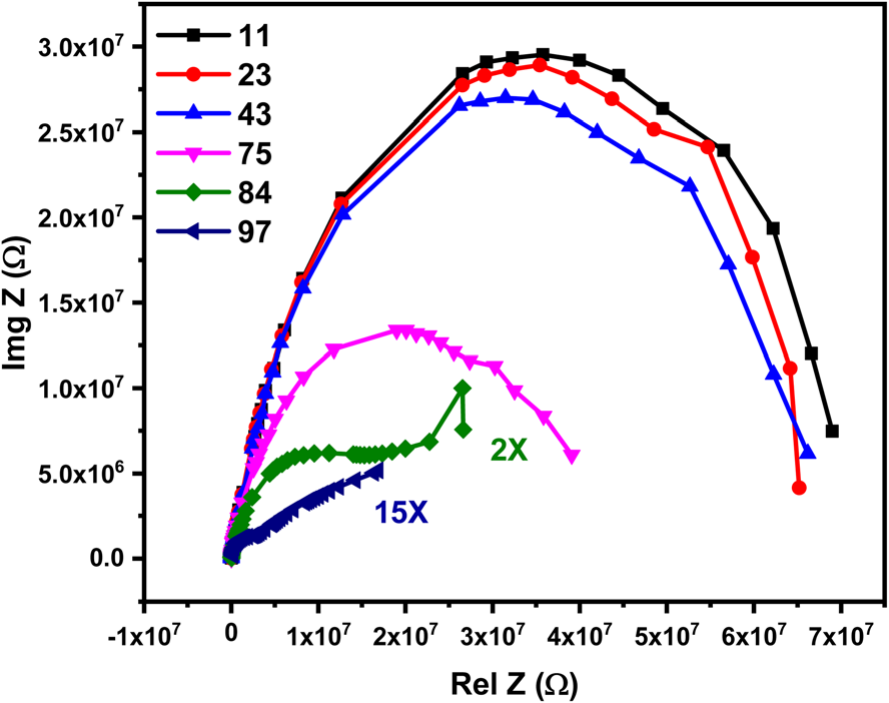
Cole–Cole diagram of the CuO–rGO-1.0 Ag humidity sensor.

The humidity sensing response of the CuO–rGO-1.0 Ag sensor was investigated using complex impedance spectroscopy (CIS) over a wide humidity range of 11% to 97% and a frequency range of 50 Hz to 5 MHz. The CIS spectra exhibits two distinct patterns (Fig. 10). At low humidity levels (≤43%), the spectra display a semicircular shape, with increasing curvature as the relative humidity rises. In contrast, the spectra show a semicircle with a pronounced tail at high humidity levels (75% to 97%).

To elucidate the underlying humidity sensing mechanism, a layer-by-layer adsorption model can be considered. At low humidity, the initial layer of water molecules is bonded to the external surface of the sensor *via* double hydrogen bonds, leading to localization of charge carriers. As the humidity increases the second layer of adsorbed water molecules dissociate into protons (H^+^) and hydroxyl ions (OH^−^). More water layers are adsorbed with further increases in humidity levels, leading to the formation of hydronium ions (H_3_O^+^) through the interaction of protons with adsorbed water molecules. The subsequent adsorbed layers interact with the generated hydroxyl group to form hydronium ions. These hydronium ions dissociate the adsorbed water layers to form additional hydronium ions. This reaction is described as a chain reaction and is known as the Grotthuss reaction. This mechanism involves the rapid hopping of protons between water molecules, pointedly boosting the ionic conductivity of the sensor.

The overall sensing mechanism can be summarized using the following equations:

7 H_2_O ↔ H^+^ + OH^−^

8 H_2_O + H^+^ ↔ H_3_O^+^

9 H_2_O + H_3_O^+^ ↔ H_3_O^+^ + H_2_O

**Fig. 11:**
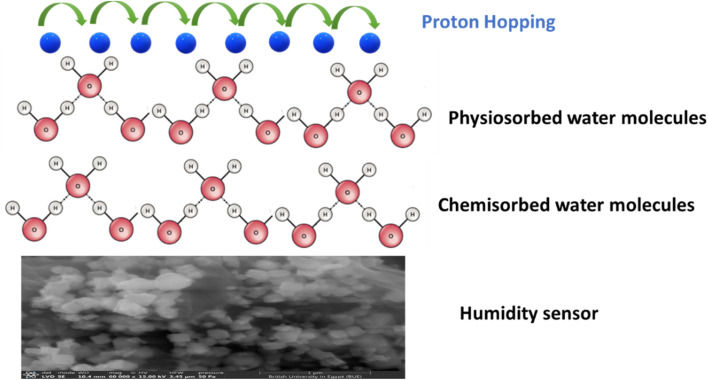
Schematic diagram of the humidity sensing mechanism.

The humidity sensing mechanism is described schematically in Fig. 11. Besides, the comparative analyses of the close reported structures directed towards the humidity sensing application is provided in Table 3.

**Table 3:** Comparison the parameters in the presented work with similar other research

Structure	Response time (s)	Recovery time (s)	Sensitivity	Relative humidity range (%)	Highest impedance/frequency, humidity	Ref.
CuO ceramic	170	60	—	30–95	—	31
CuO–rGO	2	17	—	11–98	10^6^@10 Hz, 11% RH	28
Triboelectric nanogenerators driven self-powered rGO–TiO_2_	1	5.2	—	0–95	—	21
GO/Ag (2 wt%)	8	12	—	11–97	—	32
Ag/ZnO	36	6	—	11–95	10^8^@100 Hz, 11% RH	57
CuO–rGO-Ag (1.0 wt%)	154	172	2 × 10^6^ Ω per RH	11–97	2 × 10^8^@50 Hz, 11% RH	This work

### Antibacterial study results

**Fig. 12:**
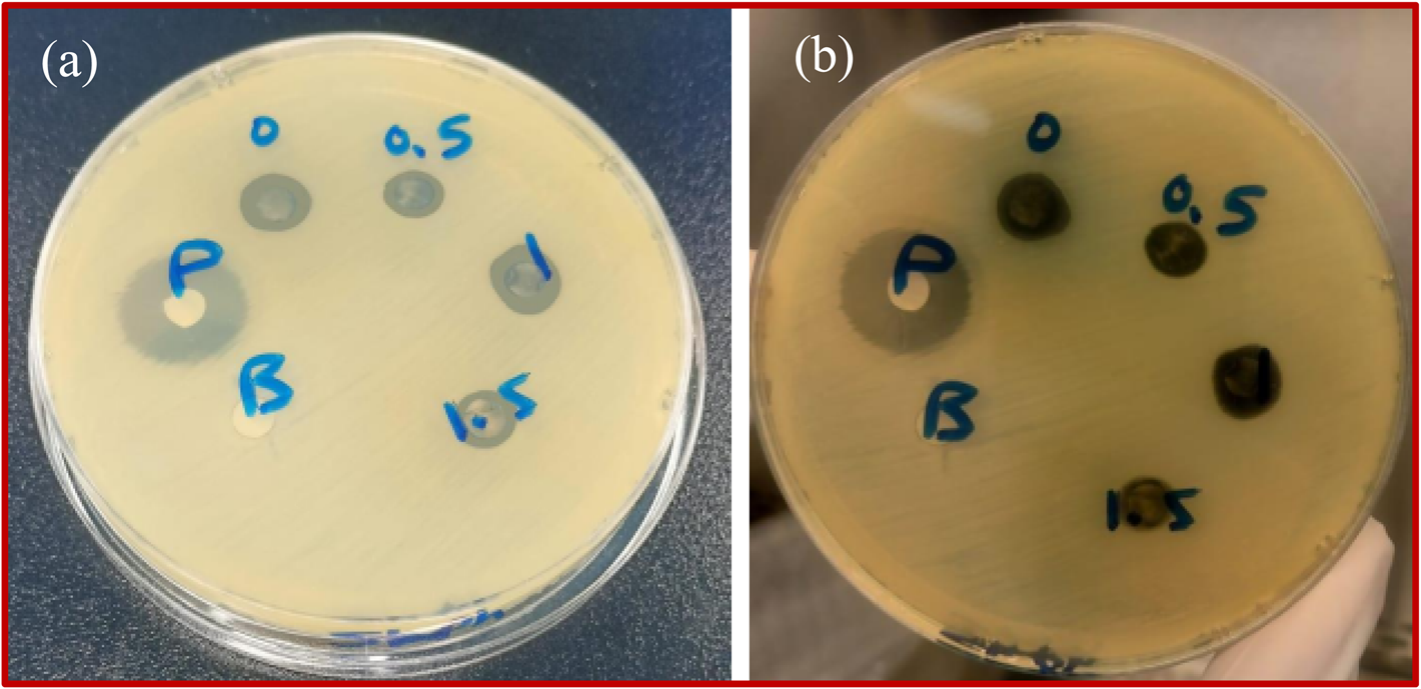
Agar well diffusion assays showing the antibacterial activity of CuO–rGO-Ag, Ag = 0, 0.5, 1.0 and 1.5 wt% Ag by viewing the inhibition zone. The antibacterial behavior against: (a) Gram-positive bacteria (*Staphylococcus*), and (b) Gram-negative bacteria (*E. coli*) is revealed.

The antibacterial exploration of the synthesized ZnO–rGO-Ag, Ag = 0, 0.5, 1.0, and 1.5 wt% is demonstrated in Fig. 12. Doped and undoped nanostructures are investigated to reveal the zone of inhibition (Table 4 and Fig. 12). As reported earlier, rGO alone does not exhibit antimicrobial activity. The bactericidal usage of rGO is possibly restricted because of the stacking in the graphene sheets by van der Waals forces.^58^ Thus, the antimicrobial tendency is ascribed to the conjugated CuO and Ag nanoparticles. Significant ZOI are displayed in Table 4 and Fig. 12. The activity against the Gram-positive bacteria is slightly lower than the activity against the Gram-negative bacteria. CuO–rGO doped Ag mildly hinders the development of the bacteria, ensuing a decrease in the amount of living bacteria. The nanostructures show increasing antibacterial activity trends against Gram-negative and Gram-positive bacteria with increasing Ag doping up to 1 wt%. The ZOI are 0.35 and 0.37 cm *versus* Gram-positive bacteria (*Staphylococcus*), and Gram-negative bacteria (*E. coli*), respectively. The functionalization of metal/metal oxide/rGO may increase the number of active species on the graphene surface potentially enabling cell inhibition through chemical modes of action.^35,58^ These residing interactions correlate with the oxidative stress promoted by the charge transfer and the emergence of reactive oxygen species. Oxidative stress is believed to be the most prominent cause for bacterial eradication.^59^ The morphology of the nanostructures contributes to the elimination of bacteria. Herein, the reduced size of Ag nanoparticles accompanied by the well distributed Cu in the graphene sheets ensures a maximum interaction and contact with the pathogen cells and provides an elevated antibacterial response.^58^ For further insights on the performance, the ELISA reader was employed for the determination of the antibacterial tendency towards Gram-positive bacteria (*Staphylococcus*) and Gram-negative bacteria (*E. coli*) (Table 5). These results of the antibacterial assay by absorbance measurements at OD 600 nm are similar to the diffusion method and validate the antibacterial features.

**Table 4:** Inhibition zone diameter of all samples against *Staphylococcus* and *E. coli*

Sample	Inhibition diameter (cm)	
	*E. coli*	*Staphylococcus*
CuO/rGO	0.23 ± 0.2	0.13 ± 0.2
CuO/rGO-0.5 M Ag	0.25 ± 0.2	0.24 ± 0.2
CuO/rGO-1.0 M Ag	0.37 ± 0.2	0.35 ± 0.2
CuO/rGO-1.5 M Ag	0.13 ± 0.2	0.19 ± 0.2
Blank	0	0
Positive	1.0	1.0

**Table 5:** Absorbance readings for three replicas at 600 nm using an ELISA reader

Sample	OD measurements of *E. coli*			OD measurements of *Staphylococcus*		
	Replica 1	Replica 2	Replica 3	Replica 1	Replica 2	Replica 3
CuO/rGO	0.258	0.267	0.265	0.256	0.255	0.251
CuO/rGO-0.5 M Ag	0.3185	0.397	0.404	0.314	0.320	0.354
CuO/rGO-1.0 M Ag	0.293	0.310	0.318	0.254	0.255	0.25
CuO/rGO-1.5 M Ag	0.321	0.319	0.340	0.333	0.333	0.315
Blank	0.069	0.06	0.062	0.053	0.054	0.057
Positive	0.8	0.84	0.84	0.7	0.71	0.75

Environmental humidity plays a critical role in the survival and proliferation of bacteria, particularly on surfaces. Numerous studies have shown that higher relative humidity (RH) can enhance the survival of certain bacterial species, while lower humidity levels may lead to desiccation and reduced viability. For instance, Tang^60^ noted that Gram-negative bacteria such as *E. coli* tend to survive longer at higher relative humidity, whereas Gram-positive bacteria may be more resilient in drier conditions due to their thicker peptidoglycan layers. Furthermore, McDevitt and Rudnick^61^ found that bacterial transmission and persistence on surfaces are powerfully impacted by ambient humidity, which influences bacterial viability and biofilm formation. For instance, it is realistic to infer that bacteria might have had better survival conditions if high RH was present during antibacterial testing, thereby providing a stringent test environment for the antibacterial efficacy of the material. Future work might be directed at systematically varying RH levels to quantify its direct impact on bacterial viability and material performance.

## Conclusion

This work prepared CuO–rGO doped with reduced amounts of Ag (0–1.5 wt%). The synthesized structures were explored through XRD, FTIR, and SEM. SEM demonstrated that the average lengths of the CuO cubic structure were 0.7 μm, 0.16 μm, 0.16 μm, and 0.12 μm for 0 M, 0.5 M, 1 M, and 1.5 M Ag doping, respectively according to Image J software. XRD results displayed the dominance of the cubic structure, and the crystallite size was determined using the Scherrer equation for the most intense peak near 36°. After characterizing the basic structural and morphological features, the humidity sensing performance was evaluated for the prepared nanostructures. The optimum testing frequency for the sensor was evaluated at 50 Hz, which was used in further experiments. The response and recovery times for the prepared sensors vary between 140–150 s between 11% RH and 97% RH. Notably, the recovery time of CuO–rGO doped with 1.5 M Ag was as low as 17 s. The sensors' repeatability was verified using four cycles between 11% and 75% RH since the impedance of the sensor returned to the same levels. Furthermore, the sensing mechanism was explained showing the interactions of the molecules and the sensor surface. This mechanism encompasses the rapid hopping of protons between water molecules, pointedly boosting the ionic conductivity of the sensor. The proposed structure can be beneficial in humidity sensing applications throughout a wide range of directions.

## Data availability

The data related to the work are all included in the manuscript.

## Author contributions

A. Elzwawy contributed to conceptualization, writing – original draft, investigation, and methodology. A. I. Abdel-Salam contributed to writing – review and editing, investigation, and formal analysis. S. Zain contributed to methodology, investigation, and formal analysis. M. Morsy contributed to conceptualization, writing – review and editing, supervision, and investigation. The authors accept the responsibility for the study conception and design, data collection, analysis and interpretation of results, and manuscript preparation. All authors agreed to the content of the manuscript.

## Conflicts of interest

The authors announce that they have no interrelated competing interests.
